# From Platelets to Preschoolers: The Pioneering Journey of Dr. Valentin Fuster in Cardiology and Global Health Education

**DOI:** 10.7759/cureus.68026

**Published:** 2024-08-28

**Authors:** Hoda Shabpiray, Alborz Sherafati, Ayda Fathollah Pour, Elizabeth Schumacher, Mani Khorsand Askari

**Affiliations:** 1 Internal Medicine, The University of Toledo College of Medicine and Life Sciences, Toledo, USA

**Keywords:** public health advocacy, medical innovation, global health initiatives, cardiovascular education, valentin fuster

## Abstract

Dr. Valentin Fuster's career, blending pioneering research with global health education, has profoundly influenced cardiology and public health. From his early life in Barcelona's medical community to groundbreaking contributions at major institutions like Mount Sinai and Harvard, Dr. Fuster has led transformative research on cardiovascular diseases and their prevention. His work spans from detailed studies on platelets and aspirin's preventative roles to innovative uses of MRI for understanding atherosclerosis. Beyond academia, he has significantly impacted public health through educational programs like the character "Dr. Ruster" on Sesame Street, advocating heart-healthy lifestyles to preschoolers worldwide. Dr. Fuster's dual focus on advanced medical research and community health initiatives exemplifies an integrated approach to tackling heart disease on multiple fronts.

## Introduction and background

Early life and influences 

**Figure 1:**
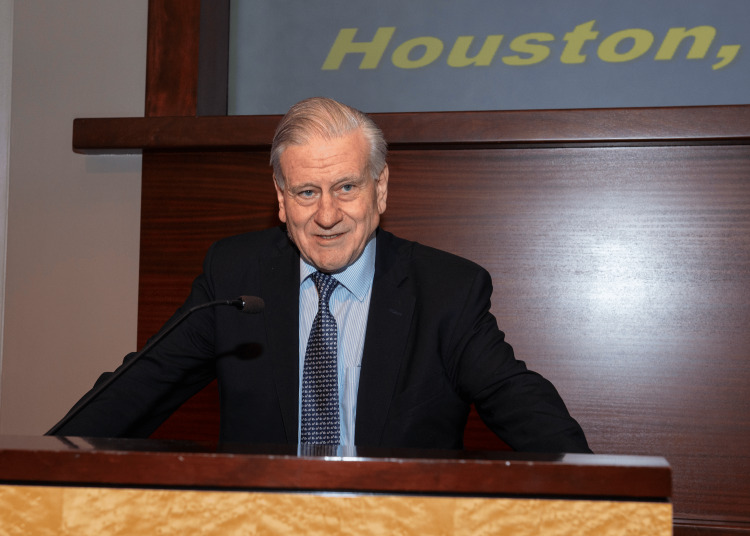
Dr. Valentine Fuster Image source: "Feb. 1, 2019 THI CGR Valentin Fuster" by Texas Heart Institute is licensed under CC BY-NC-ND 2.0.

Born in Barcelona, Spain, Dr. Valentin Fuster (Figure 1) grew up in a medical family; his father was a psychiatrist, and his brother was a neuroscientist. His maternal grandfather was the president of the University of Barcelona and helped build the city's first university hospital. He was interested in tennis, soccer, and cycling [1,2]. His competitive spirit, honed through national-level tennis, paralleled his academic pursuits, setting a foundation of discipline and resilience. He was passionate about learning languages besides Spanish, and he learned French and English through high school and medical school [1-3]. 

Significant achievements marked Dr. Fuster's academic journey. His meeting with Pedro Farreras Valenti, a pioneering physician in Spain at a tennis club, was a turning point. Dr. Farreras, impressed by Dr. Fuster's potential, became his mentor. This relationship and Dr. Fuster's dedication led to his graduation as the top student with an MD from the University of Barcelona in 1967. His thirst for knowledge and academic excellence drove him to pursue further education at the University of Edinburgh Medical School, where he earned his PhD [1,2]. 

Educational foundation 

Upon Dr. Farreras's suggestion, he studied pathology in the UK for two semesters. During his second semester, Professor Harold Sheehan showed him a slide of a blood clot full of platelets (a type of cell critical in blood clotting) from a patient who had died of a myocardial infarction (heart attack) [4]. This led Dr. Fuster to study platelet function to understand the pathogenesis of heart attack as his thesis. This led him to investigate the vessel wall's atherosclerotic disease (a disease characterized by the buildup of fats, cholesterol, and other substances in and on the artery walls, which can lead to heart attacks and strokes) and visual imaging. During this time, he conducted the first randomized study with aspirin [4]. Dr. Fuster moved to the University of Edinburgh after his semester with Professor Sheehan, where he completed his thesis on the platelet function in myocardial infarction for his PhD. His tenure there included a role as a research fellow in cardiology at the Royal Infirmary of Edinburgh, foundational years that shaped his future research trajectory [1-3]. He started a new role at Mayo Clinic, Rochester, in 1971, where he received his first National Institution of Health (NIH) grant and was promoted to the rank of professor of medicine at age 39 [3]. His research was focused on patients with Von Willebrand disease (a genetic disorder that affects blood clotting), which opened a new opportunity to study platelet function in atherosclerotic vascular disease [5]. He moved to New York City later to start his role as the chief of cardiology at Mount Sinai School of Medicine, and he was the chief of cardiology at the Massachusetts General Hospital later in his career [3]. 

## Review

Pioneering research and innovations 

Dr. Fuster's pioneering research has significantly impacted several domains of cardiovascular medicine. 

Platelets and Aspirin

His seminal work on the role of platelets in arterial disease and the preventive efficacy of aspirin has become a cornerstone in managing heart disease [6]. 

Polypill

Dr. Fuster helped develop a cardiovascular "polypill," a single pill that includes three medications typically taken separately that are effective in preventing secondary adverse cardiovascular events in people who have previously had a heart attack [4]. 

Imaging Techniques

Innovating the use of MRI to visualize arterial plaque, he enhanced our understanding of atherosclerotic disease processes, providing insights into myocardial infarction and vascular health that were previously obscured by traditional imaging techniques [7,8]. His innovative use of MRI led to using rapamycin to prevent restenosis following angioplasty. It led to the FREEDOM trial to define the best revascularization strategy for diabetic patients with multivessel coronary disease and has significantly impacted their treatment [4]. 

Academic and professional milestones 

Mayo Clinic, Mount Sinai, and Massachusetts General Hospital

From his initial role as a medical resident to becoming a professor and leading consultant in cardiology, Dr. Fuster's tenure at the Mayo Clinic laid the groundwork for his future roles. In 1981, he was appointed head of cardiology at Mount Sinai School of Medicine, a position that saw him revolutionize clinical practices and treatment methodologies [9]. He was also the chief of cardiology at Massachusetts General Hospital from 1991 to 1994 [3]. 

Global Leadership

Serving as the Mallinckrodt professor of Medicine at Harvard, director of Mount Sinai Heart, editor-in-chief at the Journal of American College of Cardiology (JACC), president of the American Heart Association (AHA), president of the World Heart Federation, and the chair of the committee on Preventing the Global Epidemic of Cardiovascular Disease at the National Academy of Science, Dr. Fuster's leadership and innovation extended to significant global policy changes and health initiatives [1,3,9]. 

Global health leadership and innovations 

Educational Initiatives

His novel approach to health education as "Dr. Ruster" on Sesame Street has impacted millions, using media to teach children preventive health measures globally [10,11]. 

World Heart Federation and Policy Advocacy

As a leader in various global organizations, Dr. Fuster has advocated for and implemented policy changes to mitigate global cardiovascular disease burdens, especially in low- and middle-income countries [11]. 

Media and public engagement 

Publications and Presentations

Dr. Valentin Fuster has leveraged his expertise as a prolific author and speaker to spread critical information about cardiovascular health. He has authored numerous scientific articles and books and delivered keynote presentations at significant global forums. His work not only shares the latest research findings but also raises awareness about the prevention and management of cardiovascular diseases, reaching a diverse audience ranging from medical professionals to public health policymakers [3].

Innovative Health Campaigns

Dr. Fuster's influence extends into popular media, notably through his advisory role with Sesame Workshop, the organization behind Sesame Street. His involvement has been pivotal in creating educational initiatives that introduce concepts of cardiovascular health to young audiences. This collaboration has led to programming that is both engaging and educational, aiming to instill healthy habits early in life. The significance of this approach lies in its ability to utilize familiar and beloved characters to communicate important health messages, making complex ideas accessible and actionable for children. Such early interventions are crucial as they can shape health behaviors and attitudes from a young age, potentially reducing the risk of cardiovascular and other lifestyle-related diseases in the future [9,10]. 

## Conclusions

Dr. Valentin Fuster's illustrious career exemplifies the profound impact a dedicated physician-scientist can have on both medicine and society. His dual focus on groundbreaking medical research and proactive public health initiatives offers a robust model for integrating scientific discovery with broad-based health improvement strategies. Through his extensive contributions to clinical research, global health policy, and innovative public health campaigns, Dr. Fuster remains one of the most influential figures in cardiology. 
